# Income-Related Inequalities in Physical and Cognitive Health Domains Over the Later Life Course: Longitudinal Evidence From the U.S. (1992–2016)

**DOI:** 10.1177/01640275231183438

**Published:** 2023-06-26

**Authors:** Mengling Cheng, Nicolas Sommet, Daniela S. Jopp, Dario Spini

**Affiliations:** 1 Swiss Centre of Expertise in Life Course Research, University of Lausanne, Lausanne, Switzerland; 2 Faculty of Social and Political Sciences, 27213 University of Lausanne, Lausanne, Switzerland; 3 87684 Institute of Social Sciences, University of Lausanne, Lausanne, Switzerland; 4 30657 Institute of Psychology, University of Lausanne, Lausanne, Switzerland; 5 Research Center for Psychology of Health, Aging and Sport Examination, University of Lausanne, Lausanne, Switzerland; 6 Life Course and Social Inequality Research Centre, University of Lausanne, Lausanne, Switzerland

**Keywords:** age-as-leveler, cumulative advantage/disadvantage, persistent inequality, health domains, longitudinal

## Abstract

This study aims to investigate changes in the income–health gradient over the later life course. We test the age-as-leveler, the cumulative advantage/disadvantage, and the persistent inequality pattern for physical and cognitive health domains, and analyze whether these patterns are gendered. We used HRS data (1992–2016) and Poisson growth curve models to predict multimorbidity (33,860 participants) as an indicator of physical health and memory (25,291 participants) as an indicator of cognitive health. We disentangled the within-participant from the between-participant effects. For multimorbidity, the income–health gradient weakened as individuals aged; whereas for memory, the income–health gradient strengthened as individuals aged. The cumulative advantage/disadvantage of higher/lower income on memory may be more pronounced among women than men. Findings were confirmed by sensitivity analyses. Findings suggest that the support for the age-as-leveler or cumulative advantage/disadvantage pattern may depend on health domains and the effect strength may depend on gender.

## Introduction

It has been established that the income–health gradient (Marmot, 2005) begins in in-utero development and persists into later life (for a review, see Corna, 2013). However, researchers are divided on the theoretical assumptions and empirical evidence regarding the evolution of the income–health gradient over the later life course. Some researchers argue that the link between income and health weakens in later life, as the impact of biological aging and selective mortality eclipses the impact of income and narrows the health gap between the rich and poor (the “age-as-leveler pattern”; O’Rand, 2009; for empirical evidence, see, e.g., Brown et al., 2016; Sieber et al., 2020). Other researchers argue that the link between income and health strengthens in later life, as the advantages of being rich and the disadvantages of being poor in a given cohort accumulate over the life course and widen the health gap between the rich and poor (the “cumulative advantage/disadvantage pattern”; Dannefer, 2020; DiPrete & Eirich, 2006; for empirical evidence, see, e.g., Boen, 2016; Veenstra & Aartsen, 2022). Finally, some other researchers argue that the link between income and health remains stable in later life, as the structures and/or processes of socioeconomic stratification persist over the life course and stabilize the health gap between the rich and poor (the “persistent inequality pattern”; Ferraro, 2011; for empirical evidence, see, e.g., Wachtler et al., 2019; Zhu & Ye, 2020). Arguably, many previous studies are limited in two ways. First, they often focus on a single generic health outcome (most often self-rated health). It is possible that the support for the age-as-leveler pattern or the cumulative advantage/disadvantage pattern varies across outcomes in different health domains (Brown et al., 2012; Xu et al., 2014). Second, they often focus on between-participant effects. Estimates of between-participant effects are more likely to be influence by selection effects than estimates of within-participant effects (Jager et al., 2020).

To address these limitations, (i) we adopted a multidimensional conceptualization of health (Hjelm, 2010) by using multiple specific outcomes from the physical and cognitive health domains and (ii) we disentangled within-participant effect of aging from between-participant effect of age over time by using longitudinal data from the Health and Retirement Study. Moreover, one remaining question is whether the age-as-leveler, the cumulative advantage/disadvantage, or the persistent inequality pattern differs between women and men. As women and men experience different levels of exposure and have differential sensitivity/vulnerability to socioeconomic determinants of health over the life course (Denton et al., 2004), these patterns may differ between women and men. To extend previous studies, we therefore investigated the role of gender in predicting these patterns.

### How the Income–Health Gradient Changes over the Later Life Course

Scholars are divided on how the income–health gradient changes with old age: proponents of the age-as-leveler pattern claim that the income–health gradient weakens with old age, proponents of the cumulative advantage/disadvantage pattern claim that the income–health gradient strengthens with old age, whereas proponenets of the persistent inequality pattern claim that the income-health gradient remains stable in old age. Importantly, the empirical evidence is also divided, with some research supporting the age-as-leveler pattern, others supporting the cumulative advantage/disadvantage pattern, and some others supporting the persistent inequality pattern.

#### Age-as-Leveler Pattern

Research supporting the age-as-leveler pattern suggests that the income–health gradient weakens with old age (O’Rand, 2009). However, age is a broad category for the observed process that needs to be revealed. Three complementary explanations are provided in the literature that can explain the age-as-leveler effect: The first explanation concerns biological aging, the second concerns selective mortality, and the third concerns social policies. According to the biological perspective, individuals from all socioeconomic backgrounds eventually experience a decline in health as they age because of the gradual accumulation of cellular defects through random molecular damage (Kirkwood, 2014). As a result, individuals with higher income lose their advantages as they age (Brown et al., 2016), experiencing a faster decline in health and eventually catching up with their lower-income counterparts (Herd, 2006). In addition, selective mortality (Dupre, 2007) may also impact on the income-health gradient in old age. This selective process is caused by mortality at younger age for the most disadvantaged. This leaves in the older adult population a more homogenous surviving population that is healthier and with higher socioeconomic status compared with the baseline population. Moreover, social policies may serve to offset disadvantages and adversities and narrow the gap in health disparities in old age by facilitating access to healthcare (Sieber et al., 2020). For instance, Medicare insurance has been shown to exert a leveling effect on disparities between socioeconomically advantaged and disadvantaged individuals in access to care and health status at age 65 (Wallace et al., 2021).

A series of empirical studies support the age-as-leveler pattern — many of which have used a single generic health outcome (most often self-rated health). For instance, studies have shown that the protective effect of higher income on self-rated health was stronger for middle-aged adults than for older adults, and that this protective effect began to wane in old age (Brown et al., 2016; Doebler & Glasgow, 2017; Li & Mutchler, 2019; Sieber et al., 2020). It is worth noting that a few existing studies that used alternative health outcomes other than self-rated health also lent support to the age-as-leveler pattern (e.g., mortality, Rehnberg et al., 2019; frailty, Van Der Linden et al., 2020).

#### Cumulative Advantage/Disadvantage Pattern

Research supporting the cumulative advantage/disadvantage pattern suggests that the income–health gradient strengthens with old age (Dannefer, 2020; DiPrete & Eirich, 2006). The theoretical assumption is that advantages and disadvantages accumulate over the life course through person–environment interactions involving individual capacity, location, resources, and context (Dannefer, 2003). Because of differential exposure to risk factors and differential access to protective resources, income-related advantages and disadvantages tend to accumulate over the life course into old age (O’Rand, 2002). Individuals with higher income are faced with less difficult life conditions, which could impact health outcomes (Lantz et al., 2005). In addition, individuals with higher income have more reserves (e.g., economic, social, or cognitive reserves) available to help overcome and recover from adverse life events or stressors, whereas individuals with lower income have fewer such reserves and are more vulnerable to such events or stressors (Cullati et al., 2018).

A series of empirical studies support the cumulative advantage/disadvantage pattern — many of which have used a single generic health outcome (again, self-rated health). For instance, studies have shown that the protective effect of higher income on self-rated health was stronger for older adults than for middle-aged adults, and that older adults with higher socioeconomic status experience a slower decline in self-rated health than those with lower socioeconomic status (Boen, 2016; Leopold, 2018, 2019; Veenstra & Aartsen, 2022). Similarly, a few existing studies that used health outcomes other than self-rated health also lent support to the cumulative advantage/disadvantage pattern (e.g., disability, Lai et al., 2022; cognition, Zeng et al., 2022).

#### Persistent Inequality Pattern

Research in line with the persistent inequality pattern suggests that the income–health gradient remains stable across the later life course (Ferraro, 2011). The theoretical explanation for this phenomenon revolves around the structures and/or processes that generate and perpetuate health inequalities early in life and throughout the life course (Mackenbach, 2017). Socioeconomic stratification, which gives differential access to resources and opportunities for achieving and maintaining a more favorable socioeconomic position, plays a crucial role in perpetuating income-related inequalities into old age (Abramson, 2015). Individuals with higher income have access to a broader range of material and non-material resources, yielding enduring health benefits (Lynch, 2020). In contrast, individuals with lower income have limited access to these resources, resulting in persistent health inequalities (Brown et al., 2016).

A series of empirical studies are consistent with the persistent inequality pattern — many of which have used a single generic health outcome (again, self-rated health). For instance, studies have shown that the protective effect of higher income on self-rated health did not differ between older adults and middle-aged adults, and that older adults with lower socioeconomic status experienced persistent inequality in self-rated health (Brown et al., 2016; Leopold, 2019; Wachtler et al., 2019; Zhu & Ye, 2020). Similarly, a few existing studies that used alternative health outcomes other than self-rated health also aligned with the persistent inequality pattern (e.g., body mass index, Boen, 2016; functional limitations, Brown, 2018).

### Theoretical and Methodological Concerns in Existing Studies

#### Limitations of Previous Studies

As seen above, many existing empirical studies are limited in that (i) they have used a single generic health outcome (often self-rated health); and/or (ii) they have focused on between-participant effects.

First, self-rated health is an omnibus construct that encompasses various aspects of physical and/or cognitive health. It is possible that the support for the age-as-leveler pattern, the cumulative advantage/disadvantage pattern, or the persistent inequality pattern varies across outcomes in different health domains (Brown et al., 2012; Xu et al., 2014), because socioeconomic indicators affect different aspects of health via different mechanisms both in terms of the onset and progression of disease (Cockerham et al., 2017). This phenomenon is unlikely to be identified using a single generic health outcome, but using multiple specific outcomes from the physical and cognitive health domains should enable to investigate the patterns of change in income-related disparities across health domains in old age.

Second, estimates of between-participant effects are more likely to be influence by selection effects than estimates of within-participant effects (Jager et al., 2020). As a result of the income–health gradient, older adults with low income tend to be underrepresented in cross-sectional surveys, either because their health status hinders them from taking part in surveys or because they have died prematurely. Therefore, older adults with low income who participate in cross-sectional surveys represent positively selected “survivors” of such selection processes, meaning that the health differences observed between older adults with high income and those with low income in these surveys may reflect this selection effect rather than the change in the income–health gradient over time per se. In contrast, in longitudinal data, the selection effect incurred by attrition could be reduced by including predictors of attrition in the main model (Henderson et al., 2000). With longitudinal data, it is possible to track how the health trajectories of older adults are shaped by income and disentangle the within-participant from the between-participant effects of income on health.

#### Remaining Question: The Role of Gender

It is known that health trajectories in later life differ between women and men (Gorman & Read, 2006). However, there is limited understanding of whether the age-as-leveler, the cumulative advantage/disadvantage, or the persistent inequality pattern differs between women and men, as many existing studies on the income–health gradient in later life have not investigated the moderating role of gender in shaping income-related health trajectories (Dannefer, 2020). It is possible that these patterns may be different between women and men, as women and men experience different levels of exposure and have differential sensitivity/vulnerability to socioeconomic determinants of health over the life course (Denton et al., 2004). In terms of different levels of exposure, women, throughout their life course, are more likely to endure a lower socioeconomic status, be subject to higher levels of exposure to risk factors, and have less access to protective factors than men (Gu et al., 2009; Read & Gorman, 2010). In terms of differential sensitivity/vulnerability, women benefit more from a higher socioeconomic status or protective factors, but are more vulnerable to risk factors like strains and stressors than men (Ferraro et al., 2009). By investigating the gender differences in the income-health gradient over time, we could better understand how gender shapes the income-related health trajectories in the later life course.

## Research Questions and Overview of the Study

In the present study, we aim to address two research questions. First, do we observe an age-as-leveler pattern, a cumulative advantage/disadvantage pattern, or a persistent inequality pattern for physical and cognitive health domains? Second, are these patterns gendered?

To address the limitations of many existing studies, we (i) used multiple specific outcomes from physical and cognitive health domains rather than a single generic health outcome and (ii) used longitudinal data of 13 waves spanning nearly 25 years to disentangle within-participant from between-participant effects over time. In addition, to extend previous studies, we investigated the role of gender in the pattern of change in income-related disparities in health over the later life course, although here also we did not formulate directional hypothesis. In the present study, we used data from the Health and Retirement Study (HRS), a nationally representative panel survey conducted biennially since 1992 that collects health data on approximately 20,000 U.S. residents aged 50 or older. We used multimorbidity as an indicator of physical health and memory as an indicator of cognitive health, which are particularly relevant to old age in the U.S. (Makovski et al., 2019; Nyberg & Pudas, 2019). We used Poisson growth curve models and disentangled the within-participant effect of aging from the between-participant effect of age. We conducted three sets of sensitivity analyses using alternative measures of health and excluding participants who died over the study period or dropped out the survey.

## Methods

### Sample

We used 13 waves of HRS data (1992–2016). The initial sample included 42,030 participants for multimorbidity and 33,542 participants for memory. To account for the longitudinal nature of the data, we treated wave-specific observations (level-1 units) as nested within participants (level-2 units). We included eligible within-participant observations based on three inclusion criteria: (i) nonmissing sociodemographic variables (multimorbidity: *n* = 41,766, 99.4%; memory: *n* = 30,043, 89.6%); (ii) age was 50 or older (multimorbidity: *n* = 40,667, 96.8%; memory sample: *n* = 29,143, 86.9%); and (iii) participation in at least two waves of observations (i.e., demonstrating within-participant variance; multimorbidity: *n* = 33,860, 80.6%; memory: *n* = 25,291, 75.4%). Our analytical sample comprises 230,101 observations from 33,860 participants for analyses using multimorbidity and 143,011 observations from 25,291 participants for analyses using memory.

### Measures

#### Equivalized Income Decile (Time-Varying)

Participants reported the sum of their own and their spouse’s income during the last calendar year (potential sources include earnings, pensions and annuities, social security, unemployment and workers’ compensation, other government transfers, capital income, and other income). To account for inflation, we converted total household income into inflation-adjusted income using the World Bank annual consumer price index (the reference year is 2010; World Bank, 2021) for each year of the survey as an inflation multiplier (i.e., we divided household income by the year-specific consumer price index). To adjust for the difference between coupled and single participants, we converted the inflation-adjusted income of the participants and their spouses into equivalized income using the OECD (2013) square root equivalence scale (i.e., we divided the inflation-adjusted income of participants and their spouses by the square root of two for coupled participants or by one for single participants). To consider the income dynamics of older adults and their spouses over the later life course in the U.S. (Dowd et al., 2010), we used the income value for each participant in each wave to create a time-varying variable of equivalized income decile (1 = *bottom 10%*; 10 = *top 10%*).

#### Multimorbidity (Time-Varying)

Participants reported whether they had been diagnosed by a doctor with any of the following seven chronic diseases: high blood pressure, diabetes, cancer, lung disease, heart disease, stroke, or arthritis (0 = *no*; 1 = *yes*). We adopted the definition of multimorbidity proposed by Marengoni et al. (2011) and counted the number of concurrent chronic diseases reported by each participant in each wave.

#### Memory (Time-Varying)

Memory was measured using immediate and delayed word recall (Park & Festini, 2016). Participants were randomly assigned one of four 10-word lists, with a different assignment over four interviews and no overlap with the word assigned to the spouse. Participants were then asked to recall these words (i) immediately (ranging from 0 to 10) and (ii) after a delay of approximately 5 minutes spent answering other questions (ranging from 0 to 10). We summed the total number of words that were recalled correctly, resulting in a combined score of immediate and delayed word recall in each wave (ranging from 0 to 20).

#### Covariates

We selected control variables based on those commonly used in previous studies examining the change in the income–health gradient over the later life course (e.g., Brown et al., 2016; Veenstra & Aartsen, 2022) as well as those known to be associated with health (e.g., marital status, see Hoffmann, 2011; race, see Li & Mutchler, 2019). We gathered the following sociodemographic variables to use them as control variables: wealth decile (from 1 = *bottom 10%* to 10 = *top 10%*), education level (1 = *less than upper secondary*, 2 = *upper secondary or vocational*, 3 = *tertiary*), gender (−0.5 = *men*, +0.5 = *women*), race (0 = *White/Caucasian*, 1 = *non-White/Caucasian*), current marital status (0 = *not married*, 1 = *married*), current working status (0 = *not working*, 1 = *working*), and household size (i.e., the number of individuals living in the household).

#### Alternative Measures of Health

We gathered alternative measures of health to use them in sensitivity analyses. For physical health domain, we used mobility (time-varying); for cognitive health domain, we used verbal skills (time-varying). For generic health outcome, we used self-rated health (time-varying). Regarding the mobility, participants reported how many of the following activities were difficult: walking one block, walking across a room, climbing one flight of stairs, getting in or out of bed, and bathing. We counted the total number of activities that were reported to be difficult (ranging from 0 to 5). Regarding the verbal skills, participants completed the tasks of object naming, president/vice president naming, and date naming. We counted the total number of naming that were correct (ranging from 0 to 8). Regarding self-rated health, participants reported their general health status (from 1 = *excellent*, 5 = *poor*).

### Analytic Strategy

#### Poisson Growth Curve Models

To estimate health trajectories over the later-life course and to consider the hierarchical structure of the HRS data, we built a series of two-level growth curve models in which wave-specific observations (level-1 units) were nested within participants (level-2 units). We built Poisson growth curve models instead of linear growth curve models (Alt et al., 2001) because each of the outcome variables (i.e., multimorbidity and memory) is a count variable that follows a Poisson distribution. We used Poisson regression rather than negative binomial regression because the overdispersion test did not reject the null hypothesis of equidispersion, for multimorbidity, *χ*^*2*^ (2, *N* = 230,101) = 86,153, *p* = 1.00; for memory, *χ*^*2*^ (2, *N* = 143,011) = 90,679, *p* = 1.00.

#### Centering Strategy to Disentangle the Within-Participant from Between-Participant Effect

To disentangle the within-participant effect of aging from the between-participant effect of age, we used the Fairbrother (2014) centering strategy and two age variables: (i) grand-mean centered mean age and (ii) person-mean centered age. To compute the *grand-mean centered mean age*, we centered each participant’s mean age across all waves on the grand mean age of all participants. This variable enables us to capture the between-participant effect of age (i.e., the effect of differences in age between participants). To compute the *person-mean centered age*, we centered each participant’s age in each wave of the survey on their individual mean age across all waves. This variable enables us to capture the within-participant effect of aging (i.e., the effect of age-related changes within a single participant over time).

#### Focal Model Equations

We regressed health outcomes on five focal predictors: (i) grand-mean centered mean age (Age_gmc_i_), (ii) income decile (Income Decile_it_), (iii) grand-mean centered mean age × income decile (to estimate whether the effect of income differs between younger and older participants), (iv) person-mean centered age (Age_cmc_it_), and (v) person-mean centered age × income decile (to estimate whether the effect of income changes as participants age) (see Eq. (1)).

(1) $$\log\left({\lambda ͅ}_{it}\right)=\left(\beta_{00}+u_{0i}\right)+\beta_{01}\times Age\_{gmc}_{i}+\beta_{10}\times Income\;{Decile}_{it}+\beta_{11}\times Age\_{gmc}_{i}\times Income\;{Decile}_{it}+\left(\beta_{12}+u_{1i}\right)\times Age\_{cmc}_{it}+\beta_{13}\times Age\_{cmc}_{it}\times Income\;{Decile}_{it}+\beta_{ij}\times {Control}_{ij}$$

Where *Y*_it_ ∼ Poisson (*λ*_it_); *Y*_it_ is the outcome, which follows a Poisson distribution; *i* = 1, 2, …, *N* (participants); *t* = 1, 2, …, 13 (waves); *β*_ij_ × Control_ij_ represents the vector of control variables; *u*_0i_ represents the participant-level residuals; and *u*_1i_ represents the random slope of age. Decomposition of the interaction term Age_gmc_i_ × Income Decile_it_ enables to compare the effect of income decile between younger participants and older participants, and decomposition of the interaction term Age_cmc_it_ × Income Decile_it_ enables to compare the effect of income decile as participants age over the later life course. The age-as-leveler pattern corresponds to a weakening effect of higher income with old age, the cumulative advantage/disadvantage pattern to a strengthening effect of higher income with old age, and the persistent inequality pattern to a main protective effect of higher income with a null interaction with old age.

To test the role of gender, we included two-way and three-way interactions with gender in our model (see Eq. (2)).

(2) $$\log\left({\lambda ͅ}_{it}\right)=\left(\beta_{00}+u_{0i}\right)+\beta_{01}\times Age\_{gmc}_{i}+\beta_{10}\;\times \;Income\;{Decile}_{it}+\beta_{11}\times Age\_{gmc}_{i}\times Income\;{Decile}_{it}+\left(\beta_{12}+u_{1i}\right)\times Age\_{cmc}_{it}+\beta_{13}\times Age\_{cmc}_{it}\;\times \;Income\;{Decile}_{it}+\beta_{02}\;\times \;Age\_{gmc}_{i}\times {Gender}_{i}+\beta_{14}\times Age\_{cmc}_{it}\;\times \;{Gender}_{i}+\beta_{15}\;\times \;Income\;{Decile}_{it}\times {Gender}_{i}+\beta_{16}\times Age\_{gmc}_{i}\times Income\;\;{Decile}_{it}\times {Gender}_{i}+\beta_{17}\times Age\_{cmc}_{it}\times Income\;{Decile}_{it}\times {Gender}_{i}+\beta_{ij}\times {Control}_{ij}$$

We ran the Poisson growth curve models described above using the glmer function from the lme4 package (version 1.1–26) (Bates et al., 2015) in R (version 4.0.2). The R script to reproduce our findings is available via the Open Science Framework (OSF): https://osf.io/8wcey/?view_only=f04a4c585bd3496db340b9399fce5517.

## Results

Our final sample comprises 230,101 observations from 33,860 participants for analyses using multimorbidity. Our final sample comprises 143,011 observations from 25,291 participants for analyses using memory. The sample characteristics are reported in Table 1.

**Table 1.** Sample Characteristics.

	Multimorbidity Sample (33,860 Participants)	Memory Sample (25,291 Participants)
Women (%)	56.25	57.89
Age (years) [mean (SD)]	66.6 (10.2)	64.2 (9.2)
White (%)	75.75	76.73
Currently married (%)	67.42	68.68
Currently not working (%)	47.10	43.04
Household size (persons) [mean (SD)]	2.5 (1.3)	2.5 (1.3)
Equivalized annual income (in 2010 USD) [mean (SD)]	52,715 (86,086)	54,749 (72,749)
Household wealth (in 2010 USD) [mean (SD)]	85,946 (394,375)	90,336 (383,950)
Educational levels (%)		
less than upper secondary	26.00	22.96
upper secondary or vocational	33.67	34.39
tertiary	40.33	42.65
Multimorbidity (%)	43.33	-
Memory score [mean (SD)]	-	9.5 (3.0)

### Main Analyses

#### Changes in the Income–Health Gradient over the Later Life Course

##### Multimorbidity

Our analyses using multimorbidity suggested that although the effect of income decile remained significant even in old age, the income–health gradient weakened as individuals aged (both between-and within-participant; see Table 2, left column).

**Table 2.** Effect of Income on Multimorbidity and Memory as a Function of Age among Older Adults in the U.S.

	Multimorbidity		Memory	
	IRRs	95% CI	IRRs	95% CI
Grand-mean centered mean age	1.14***	1.13–1.15	0.86***	0.86–0.87
Person-mean centered age	1.87***	1.86–1.89	0.83***	0.83–0.83
Income decile (1 = *bottom 10%*, 10 = *top 10%*)	0.89***	0.88–0.91	1.09***	1.08–1.10
Grand-mean centered mean age × income decile	1.12***	1.10–1.14	1.05***	1.04–1.06
Age (50 years old)	0.75***	0.72–0.78	1.02*	1.01–1.03
Age (−1 SD)	0.81***	0.79–0.83	1.05***	1.04–1.06
Age (+1 SD)	0.99	0.96–1.02	1.13***	1.12–1.15
Age (+2 SD)	1.09***	1.05–1.14	1.18***	1.16–1.20
Person-mean centered age × income decile	1.17***	1.14–1.19	1.13***	1.11–1.15
Panel waves (−2 SD)	0.77***	0.75–0.80	0.99	0.98–1.01
Panel waves (−1 SD)	0.84***	0.81–0.86	1.04***	1.03–1.05
Panel waves (+1 SD)	0.97*	0.95–0.99	1.15***	1.14–1.16
Panel waves (+2 SD)	1.05***	1.02–1.08	1.21***	1.19–1.23
Wealth decile (1* = bottom 10%*, 10 = *top 10%*)	0.73***	0.71–0.76	1.12***	1.11–1.14
Upper secondary or vocational education	0.96***	0.94–0.98	1.16***	1.15–1.17
Tertiary education	0.90***	0.88–0.92	1.27***	1.25–1.28
Gender (−0.5 = *men*, +0.5 = *women*)	0.96***	0.95–0.98	1.13***	1.12–1.13
Race (0 = *White/Caucasian*, 1 = *non-White/Caucasian*)	1.04***	1.02–1.06	0.90***	0.90–0.91
Current marital status (0 = *not married*, 1 = *married*)	0.99	0.98–1.01	1.02***	1.02–1.03
Current working status (0 = *not working*, 1 = *working*)	0.87***	0.86–0.88	1.01***	1.01–1.02
Household size	1.00	0.99–1.00	0.99***	0.99–0.99
Number of participants	33,860		25,291	
Number of observations	230,101		143,011	

*Note.* IRRs = Incidence Rate Ratios.

The effect of income and wealth refers to the comparison between the bottom 10% and the top 10%.

^*^p < .05. ^**^p < .01. ^***^p < .001.

##### Between-Participant Effect

**Figure 1.**
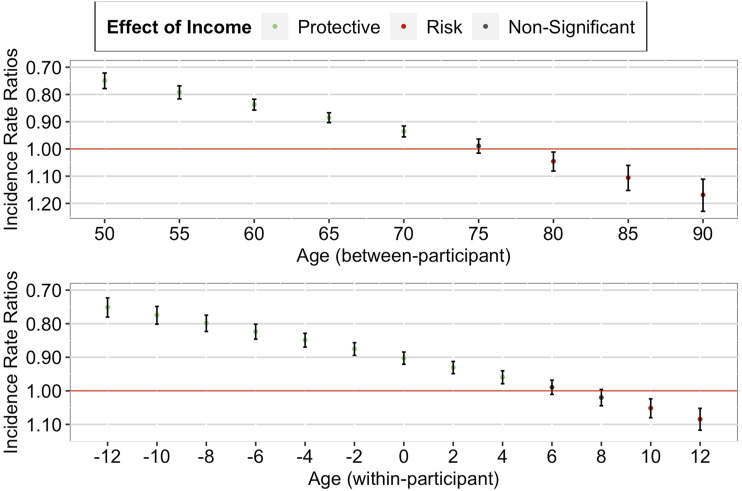
*Age-as-Leveler Effects of Income on Multimorbidity among Older Adults in the U.S*. *Note.* Red lines correspond to a null effect; error bars represent 95% CIs. The *x*-axis in the lower panel corresponds to the person-mean centered age, where zero refers to the mean age of the participant across all waves, negative numbers refer to the participant’s age in earlier waves, and positive numbers refer to the participant’s age in later waves.

Our between-participant analysis found a significant positive interaction effect between income decile and grand-mean centered mean age, IRR = 1.12, 95% CI [1.10, 1.14], *p* < .001. As seen in Figure 1 (upper panel), a further decomposition of the interaction effect suggested that the between-participant protective effect of higher income against multimorbidity was stronger for individuals in their 50s than for individuals in their 60s. Importantly, although this protective effect decreased, it remained significant until age 75 (+1 SD).

##### Within-Participant Effect

Similarly, our within-participant analysis found a significant positive interaction effect between income decile and person-mean centered age, IRR = 1.17, 95% CI [1.14, 1.19], *p* < .001. As seen in Figure 1 (lower panel), a further decomposition of the interaction effect suggested that the within-participant protective effect of higher income against multimorbidity weakened as the individual aged (from just joining the panel to later panel waves). Importantly, although this protective effect decreased, it remained significant until advanced age of the individual (+1 SD).

##### Memory

Our analyses using memory suggested that although the effect of income decile remained significant even in old age, the income–health gradient strengthened as individuals aged (both between- and within-participant; see Table 2, right column).

##### Between-Participant Effect

**Figure 2.**
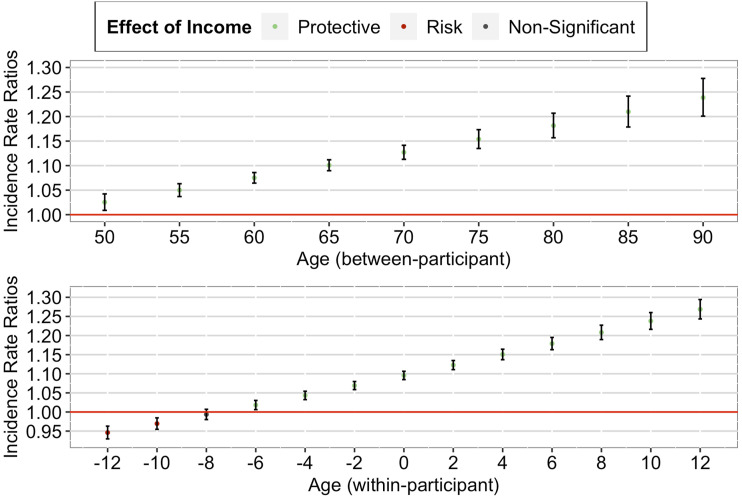
*Cumulative Advantage/Disadvantage Effects of Income on Memory among Older Adults in the U.S. Note.* Red lines correspond to a null effect; error bars represent 95% CIs. The *x*-axis in the lower panel corresponds to the person-mean centered age, where zero refers to the mean age of the participant across all waves, negative numbers refer to the participant’s age in earlier waves, and positive numbers refer to the participant’s age in later waves.

Our between-participant analysis found a significant positive interaction effect between income decile and grand-mean centered mean age, IRR = 1.05, 95% CI [1.04, 1.06], *p* < .001. As seen in Figure 2 (upper panel), a further decomposition of the interaction effect suggested that the between-participant protective effect of higher income on memory was strongest for individuals in their 80s, followed by individuals in their 70s and 60s, and was weakest for individuals in their 50s. Importantly, this protective effect remained significant over the later life course.

##### Within-Participant Effect

Similarly, our within-participant analysis found a significant positive interaction effect between income decile and person-mean centered age, IRR = 1.13, 95% CI [1.11, 1.15], *p* < .001. As seen in Figure 2 (lower panel), a further decomposition of the interaction effect suggested that the within-participant protective effect of higher income on memory strengthened as the individual aged (from just joining the panel to later panel waves). Importantly, this protective effect started when the individual entered old age and remained significant over the later life course of the individual.

#### Gender as Moderator

**Figure 3.**
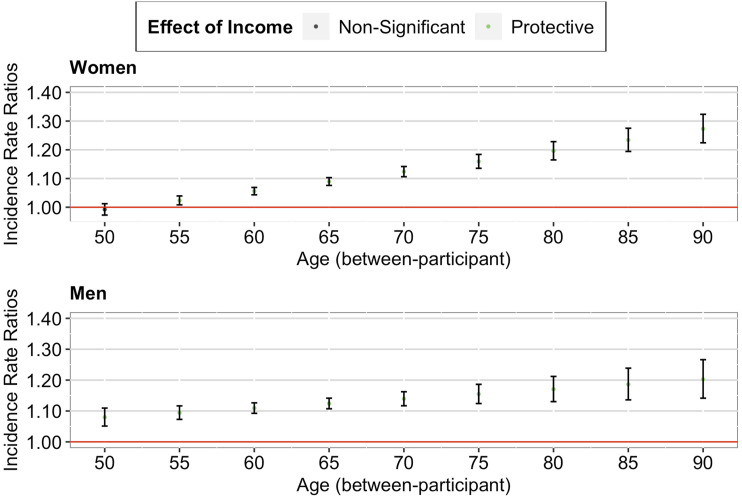
*Between-Participant Cumulative Advantage/Disadvantage Effects of Income on Memory among Older Adults in the U.S. (by Gender)*. *Note.* Red lines correspond to a null effect; error bars represent 95% CIs.

**Figure 4.**
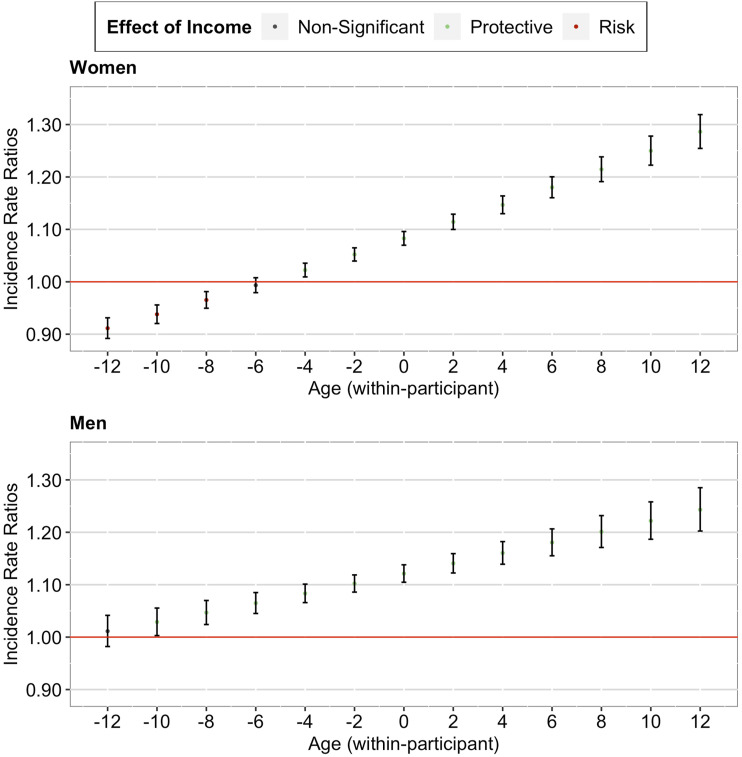
*Within-Participant Cumulative Advantage/Disadvantage Effects of Income on Memory among Older Adults in the U.S. (by Gender*). *Note.* Red lines correspond to a null effect; error bars represent 95% CIs. The *x*-axis in both panels corresponds to the person-mean centered age, where zero refers to the mean age of the participant across all waves, negative numbers refer to the participant’s age in earlier waves, and positive numbers refer to the participant’s age in later waves.

Our analyses including the gender interaction terms revealed three findings (see Supplementary Table 1). First, the observed effects on multimorbidity and memory remained significant (both between- and within-participant). Second, the observed effects on multimorbidity did not differ between women and men (both between- and within-participant). Third, the observed effects on memory tended to be more pronounced among women than men (between-participant, see Figure 3; within-participant, see Figure 4).

### Sensitivity Analyses

We conducted three sets of sensitivity analyses. The first set of sensitivity analyses used mobility (physical health domain) and verbal skills (cognitive health domain). We obtained generally consistent results with those of the main analyses. On the one hand, as shown in Supplementary Table 2, we observed similar effects on mobility (compared to multimorbidity) and similar effects on verbal skills (compared to memory), both between- and within-participant. On the other hand, as shown in Supplementary Table 3, the effects on mobility did not differ between women and men (both between- and within-participant), whereas the effects on verbal skills were significant only among women (between-participant). The second set of sensitivity analyses used self-rated health (a generic health outcome). We found that the effect of higher income on self-rated health weakened as individuals aged (both between- and within-participant; Supplementary Table 4), and that this effect did not differ between women and men (both between- and within-participant; Supplementary Table 5). The third set of sensitivity analyses excluded participants who either died over the study period or dropped out the survey. We obtained generally consistent results with those of the main analyses. The effects on multimorbidity and memory remained the same (Supplementary Table 6) and the effects on memory were more pronounced among women than men (Supplementary Table 7).

## Discussion

In this study, we used multiple specific physical and cognitive health outcomes and longitudinal data of 13 waves spanning nearly 25 years to test the age-as-leveler pattern, the cumulative advantage/disadvantage pattern, and the persistent inequality pattern for physical and cognitive health domains, and analyze whether these patterns are gendered.

Our study yields two main findings. First, our between-participant and within-participant results suggest that the support for the age-as-leveler pattern or the cumulative advantage/disadvantage pattern tends to vary across physical and cognitive health domains. Regarding physical health, our between-participant and within-participant results support the age-as-leveler pattern. Our results are in line with the findings of previous cross-sectional studies (e.g., Li & Mutchler, 2019) and the few previous longitudinal studies that are available (e.g., Brown et al., 2016; Rehnberg et al., 2019; Van Der Linden et al., 2020). Given that the age-as-leveler pattern was observed not only between participants but also within participants, and that this pattern was robust to sensitivity analyses excluding participants who died or dropped out during follow-up, our study suggests that the selection-based explanations are less likely to account for this pattern. One possible explanation is the compression of morbidity in later life (Payne, 2022). A higher income helps older adults maintain good health, and the period of morbidity is compressed into advanced age, after which they experience a fast decline in physical health. Another possible explanation is age-related health deterioration (Hoffmann, 2011). In later life, the biological effects of aging become more predictive of physical health than socioeconomic indicators.

Regarding cognitive health, our between-participant and within-participant results support the cumulative advantage/disadvantage pattern. Our results are in line with the findings of previous cross-sectional studies (e.g., Andel et al., 2017) and the few previous longitudinal studies that are available (e.g., Leopold, 2019; Xu et al., 2017; Zeng et al., 2022). One possible explanation is that initial advantages lead to additional advantages and initial disadvantages lead to further disadvantages over time (Crystal et al., 2016). Individuals with higher income accumulate advantages over the life course, whereas individuals with lower income accumulate disadvantages in cognition, which widens the disparities in cognitive health. Another possible explanation is the cognitive reserves accumulated over the life course (Cullati et al., 2018). Individuals with higher income are more exposed to cognitively stimulating activities in their life than individuals with lower income, which widens the disparities in cognitive health.

However, our between-participant and within-participant results suggest that neither the age-as-leveler nor the cumulative advantage/disadvantage pattern is mutually exclusive with the persistent inequality pattern. Although we found evidence of interactions, our between-participant and within-participant results show that the protective effect of higher income on physical and cognitive health remains significant over the later life course, which suggests a *partially* persistent inequality pattern. In other words, despite the age-as-leveler pattern in physical health and cumulative advantage/disadvantage pattern in cognitive health, income-related inequalities in physical and cognitive health neither fully emerge nor completely disappear in old age.

Second, we find that the cumulative advantage/disadvantage pattern may be gendered. The cumulative advantage/disadvantage effects of income on cognitive health tend to be more pronounced among women than men. Our results are in line with those of the few studies that stratify by gender (Hu et al., 2020; Lee & Park, 2019). Not only do individuals with lower income accumulate their disadvantage in cognitive health over time, but women do as well, meaning that as time passes, women with lower income are likely to find themselves less cognitively healthy than their men counterparts. One possible explanation for the stronger cumulative advantage/disadvantage effects of income on cognitive health among women is that in the inequality accumulation process, women tend to have more exposure to risk factors and fewer resources and accumulate disadvantage more easily than men (Ferraro et al., 2009).

### Limitations and Future Research

Three limitations need to be acknowledged. First, our sample was from the U.S., a Western and industrialized country. We chose to work with the HRS data because it is the longest-running panel data on older adults in the U.S. However, this means that our results cannot be generalized to other countries and that replication studies using samples from countries with different characteristics are needed. Second, multimorbidity was based on self-reported chronic diseases collected in interviews rather than based on clinical records. Despite the plausible measurement error, chronic disease data from health interview surveys have been proven to show acceptable reliability and validity (Beckett et al., 2000) and have been used in studies on the change in the income–health gradient over the later life course (e.g., Brown et al., 2012). Third, we did not specifically examine the role of race/ethnicity in predicting the changes in the income-health gradient over the later life course, as it was deemed beyond the scope of our study. Future research needs to consider the intersectionality of race/ethnicity, age, gender, and socioeconomic status to better understand how these factors shape the health trajectories over time.

### Conclusion

Our findings suggest that in the U.S., as time passes whether the protective effect of higher income on health tapers off or burgeons tends to vary across physical and cognitive health domains, and that the strength of the effect may differ between women and men.

## Supplemental Material

Supplemental Material - Income-Related Inequalities in Physical and Cognitive Health Domains Over the Later Life Course: Longitudinal Evidence From the U.S. (1992–2016) Click here for additional data file. Supplemental Material for Income-Related Inequalities in Physical and Cognitive Health Domains Over the Later Life Course: Longitudinal Evidence From the U.S. (1992–2016) by Mengling Cheng, Nicolas Sommet, Daniela Jopp and Dario Spini in Research on Aging
