# Supplementary material for: Income-Related Inequalities in Physical and Cognitive Health Domains Over the Later Life Course: Longitudinal Evidence From the U.S. (1992–2016)
Source: Res Aging. 2023 Jun 26;46(1):59–71. doi: 10.1177/01640275231183438 (PMC10666496; doi:10.1177/01640275231183438)
Supplement: Supplemental Material - Income-Related Inequalities in Physical and Cognitive Health Domains Over the Later Life Course: Longitudinal Evidence From the U.S. (1992–2016) [file sj-pdf-1-roa-10.1177_01640275231183438.pdf]

Supplementary Table 1

Effects of Income on Multimorbidity and Memory as a Function of Age among Older Adults in the U.S. (by Gender)

	Multimorbidity		Memory	
	IRRs	95% CI	IRRs	95% CI
Income decile (1 = *bottom 10%*, 10 = *top 10%*)	0.90***	0.89–0.92	1.10***	1.09–1.11
Gender (-0.5 = *men*, +0.5 = *women*)	1.01	0.99–1.03	1.13***	1.13–1.14
Grand-mean centered mean age × income decile	1.11***	1.09–1.14	1.05***	1.03–1.06
Person-mean centered age × income decile	1.14***	1.11–1.17	1.12***	1.11–1.14
(Grand-mean centered mean age × income decile) × gender	1.02	0.98–1.06	1.04**	1.01–1.06
For men	/	/	1.03**	1.01–1.05
For women	/	/	1.06***	1.05–1.08
(Person-mean centered age × income decile) × gender	1.05	1.00–1.10	1.06***	1.03–1.09
For men	/	/	1.09***	1.06–1.12
For women	/	/	1.15***	1.14–1.17
Number of participants	33,860		25,291	
Number of observations	230,101		143,011	

Note. IRRs = Incidence Rate Ratios.

The effect of income refers to the comparison between the bottom 10% and the top 10%.

Adjusted for wealth, education, race, current marital status, current working status, and household size.

$^{*}p < .05$. $^{**}p < .01$. $^{***}p < .001$

Supplementary Table 2

Effect of Income on Mobility and Verbal Skills as a Function of Age among Older Adults in the U.S.

	Mobility		Verbal skills	
	IRRs	95% CI	IRRs	95% CI
Income decile (1 = *bottom 10%*, 10 = *top 10%*)	0.68***	0.65–0.71	1.05***	1.04–1.07
Grand-mean centered mean age × income decile	1.16***	1.12–1.22	1.04***	1.02–1.05
Age (50 years old)	0.54***	0.49–0.58	1.01	0.99–1.02
Age (-1 SD)	0.59***	0.55–0.63	1.03***	1.02–1.04
Age (+1 SD)	0.78***	0.74–0.82	1.08***	1.06–1.09
Age (+2 SD)	0.89**	0.82–0.97	1.10***	1.08–1.13
Person-mean centered age × income decile	1.29***	1.20–1.39	1.11***	1.09–1.13
Panel waves (-2 SD)	0.53***	0.48–0.58	0.97**	0.96–0.99
Panel waves (-1 SD)	0.60***	0.56–0.64	1.01*	1.01–1.03
Panel waves (+1 SD)	0.78***	0.74–0.83	1.10***	1.09–1.12
Panel waves (+2 SD)	0.89**	0.83–0.97	1.15***	1.13–1.17
Number of participants	30,103		18,512	
Number of observations	187,94		103,228	

Note. IRRs = incidence rate ratios.
The effect of income refers to the comparison between the bottom 10% and the top 10%.
Adjusted for wealth, education, gender, race, current marital status, current working status, and household size.
$^{*}p < .05$. $^{**}p < .01$. $^{***}p < .001$

Supplementary Table 3

Effects of Income on Mobility and Verbal Skills as a Function of Age among Older Adults in the U.S. (by Gender)

	Mobility		Verbal skills	
	IRRs	95% CI	IRRs	95% CI
Income decile (1 = *bottom 10%*, 10 = *top 10%*)	0.67***	0.64–0.70	1.05***	1.04–1.07
Gender (-0.5 = *men*, +0.5 = *women*)	1.45***	1.38–1.52	1.01**	1.01–1.01
Grand-mean centered mean age × income decile	1.18***	1.13–1.24	1.03***	1.01–1.04
Person-mean centered age × income decile	1.26***	1.16–1.36	1.11***	1.09–1.13
(Grand-mean centered mean age × income decile) × gender	0.93	0.85–1.03	1.04*	1.01–1.07
For men	/	/	1.01	0.99–1.03
For women	/	/	1.05***	1.03–1.07
(Person-mean centered age × income decile) × gender	0.94	0.80–1.09	1.03	0.99–1.07
For men	/	/	/	/
For women	/	/	/	/
Number of participants	30,103		18,512	
Number of observations	187,945		103,228	

Note. IRRs = Incidence Rate Ratios.

The effect of income refers to the comparison between the bottom 10% and the top 10%.

Adjusted for wealth, education, race, current marital status, current working status, and household size.

$^{*}p < .05$. $^{**}p < .01$. $^{***}p < .001$

Supplementary Table 4

Effect of Income on Self-Rated Health as a Function of Age among Older Adults in the U.S.

	Self-rated health	
	IRRs	95% CI
Income decile (1 = *bottom 10%*, 10 = *top*	0.83***	0.82–0.84
Grand-mean centered mean age × income	1.06***	1.04–1.07
Age (50 years old)	0.76***	0.75–0.78
Age (-1 SD)	0.79***	0.78–0.81
Age (+1 SD)	0.88***	0.86–0.89
Age (+2 SD)	0.92***	0.90–0.94
Person-mean centered age × income decile	1.08***	1.07–1.10
Panel waves (-2 SD)	0.77***	0.76–0.79
Panel waves (-1 SD)	0.80***	0.79–0.82
Panel waves (+1 SD)	0.87***	0.86–0.89
Panel waves (+2 SD)	0.91***	0.89–0.93
Number of participants	33,878	
Number of observations	230,239	

Note. IRRs = incidence rate ratios.
The effect of income refers to the comparison between the bottom 10% and the top 10%.
Adjusted for wealth, education, gender, race, current marital status, current working status, and household size.
$^{*}p < .05$. $^{**}p < .01$. $^{***}p < .001$

Supplementary Table 5

Effects of Income on Self-Rated Health as a Function of Age among Older Adults in the U.S. (by Gender)

	Self-rated health	
	IRRs	95% CI
Income decile (1 = *bottom 10%*, 10 = *top 10%*)	0.84***	0.83–0.85
Gender (-0.5 = *men*, +0.5 = *women*)	0.98***	0.97–0.98
Grand-mean centered mean age × income decile	1.06***	1.05–1.07
Person-mean centered age × income decile	1.08***	1.07–1.10
(Grand-mean centered mean age × income decile) × gender	0.99	0.97–1.02
For men	/	/
For women	/	/
(Person-mean centered age × income decile) × gender	0.98	0.94–1.01
For men	/	/
For women	/	/
Number of participants	33,878	
Number of observations	230,239	

Note. IRRs = Incidence Rate Ratios.

The effect of income refers to the comparison between the bottom 10% and the top 10%.

Adjusted for wealth, education, race, current marital status, current working status, and household size.

$^{*}p < .05$. $^{**}p < .01$. $^{***}p < .001$

Supplementary Table 6

Effect of Income on Multimorbidity and Memory as a Function of Age in Samples Excluding Deaths and Dropout

	**Sample excluding deaths**				**Sample excluding dropout**			
	Multimorbidity		Memory		Multimorbidity		Memory	
	IRRs	95% CI	IRRs	95% CI	IRRs	95% CI	IRRs	95% CI
Grand-mean centered mean age	1.13***	1.12–1.15	0.90***	0.90–0.91	1.13***	1.12–1.14	0.86***	0.86–0.87
Person-mean centered age	1.94***	1.92–1.95	0.86***	0.86–0.86	1.86***	1.85–1.87	0.83***	0.83–0.83
Income decile (1 = *bottom 10%*, 10 = *top 10%*)	0.88***	0.86–0.90	1.08***	1.07–1.10	0.90***	0.88–0.92	1.09***	1.08–1.10
Grand-mean centered mean age × income decile	1.14***	1.10–1.17	1.04***	1.02–1.05	1.11***	1.09–1.14	1.05***	1.04–1.06
Age (50 years old)	0.76***	0.72–0.80	1.04***	1.02–1.05	0.75***	0.72–0.78	1.02*	1.00–1.03
Age (-1 SD)	0.80***	0.78–0.83	1.06***	1.05–1.08	0.81***	0.79–0.84	1.05***	1.04–1.06
Age (+1 SD)	0.96*	0.94–0.99	1.11***	1.10–1.12	0.99	0.97–1.02	1.13***	1.12–1.14
Age (+2 SD)	1.06*	1.01–1.11	1.14***	1.12–1.16	1.10***	1.05–1.14	1.18***	1.15–1.20
Person-mean centered age × income decile	1.18***	1.15–1.22	1.10***	1.08–1.11	1.16***	1.13–1.19	1.13***	1.11–1.15
Panel waves (-2 SD)	0.74***	0.71–0.78	0.99	0.98–1.01	0.77***	0.74–0.79	0.99	0.97–1.01
Panel waves (-1 SD)	0.82***	0.79–0.85	1.04***	1.03–1.06	0.83***	0.81–0.86	1.03***	1.02–1.04
Panel waves (+1 SD)	0.99	0.97–1.03	1.15***	1.13–1.16	0.98	0.96–1.01	1.16***	1.14–1.17
Panel waves (+2 SD)	1.10***	1.06–1.14	1.20***	1.18–1.23	1.07***	1.04–1.10	1.23***	1.21–1.25
Wealth decile (*1 = bottom 10%*, 10 = *top 10%*)	0.69***	0.66–0.72	1.10***	1.09–1.12	0.74***	0.72–0.76	1.12***	1.11–1.14
Upper secondary or vocational education	0.99	0.96–1.02	1.15***	1.14–1.16	0.96***	0.94–0.98	1.16***	1.15–1.17
Tertiary education	0.93***	0.90–0.96	1.25***	1.24–1.27	0.90***	0.88–0.92	1.27***	1.26–1.28
Gender (-0.5 = *men*, +0.5 = *women*)	0.97**	0.95–0.99	1.12***	1.11–1.13	0.96***	0.95–0.98	1.13***	1.12–1.14
Race (0 = *White/Caucasian*, 1 = *non-White/Caucasian*)	1.08***	1.05–1.10	0.91***	0.90–0.91	1.03***	1.01–1.05	0.90***	0.90–0.91
Current marital status (0 = *not married*, 1 = *married*)	1.00	0.99–1.02	1.02***	1.02–1.03	0.99	0.98–1.01	1.02***	1.02–1.03
Current working status (0 = *not working*, 1 = *working*)	0.90***	0.89–0.91	1.02***	1.01–1.02	0.87***	0.87–0.88	1.01***	1.01–1.02
Household size	0.99*	0.99–1.00	0.99***	0.99–0.99	1.00	0.99–1.00	0.99***	0.99–0.99
Number of participants	20,329		16,679		32,032		24,567	
Number of observations	150,785		101,039		222,711		140,186	

Note. IRRs = Incidence Rate Ratios.
The effect of income and wealth refers to the comparison between the bottom 10% and the top 10%.
$^{*}p < .05$. $^{**}p < .01$. $^{***}p < .001$

Supplementary Table 7

Effect of Income on Multimorbidity and Memory as a Function of Age in Samples Excluding Deaths and Dropout (by Gender)

	**Sample excluding deaths**				**Sample excluding dropout**			
	Multimorbidity		Memory		Multimorbidity		Memory	
	IRRs	95% CI	IRRs	95% CI	IRRs	95% CI	IRRs	95% CI
Income decile (1 = *bottom 10%*, 10 = *top 10%*)	0.89***	0.87–0.92	1.09***	1.08–1.10	0.91***	0.89–0.93	1.10***	1.08–1.11
Gender (-0.5 = *men*, +0.5 = *women*)	1.04**	1.01–1.07	1.12***	1.12–1.13	1.01	0.99–1.03	1.14***	1.13–1.14
Grand-mean centered mean age × income decile	1.13***	1.09–1.16	1.04***	1.02–1.05	1.11***	1.09–1.14	1.05***	1.03–1.06
Person-mean centered age × income decile	1.16***	1.13–1.20	1.09***	1.07–1.11	1.14***	1.11–1.16	1.12***	1.11–1.14
(Grand-mean centered mean age × income decile) × gender	1.04	0.97–1.11	1.01	0.98–1.04	1.02	0.98–1.06	1.04***	1.01–1.06
For men	/	/	/	/	/	/	1.03***	1.01–1.05
For women	/	/	/	/	/	/	1.07***	1.06–1.09
(Person-mean centered age × income decile) × gender	1.04	0.99–1.11	1.05**	1.02–1.08	1.05	1.00–1.10	1.06***	1.03–1.09
For men	/	/	1.06***	1.04–1.09	/	/	1.09***	1.06–1.11
For women	/	/	1.12***	1.10–1.14	/	/	1.15***	1.14–1.17
Number of participants	20,329		16,679		32,032		24,567	
Number of observations	150,785		101,039		222,711		140,186	

Note. IRRs = Incidence Rate Ratios.

The effect of income and wealth refers to the comparison between the bottom 10% and the top 10%.

Adjusted for wealth, education, race, current marital status, current working status, and household size.

$^{*}p < .05$. $^{**}p < .01$. $^{***}p < .001$
